# Exploring the prevalence and factors associated with fatigue and quality of life in patients with Ankylosing Spondylitis

**DOI:** 10.1192/j.eurpsy.2023.2022

**Published:** 2023-07-19

**Authors:** A. Feki, Y. Mejdoub, I. Sellami, I. Mnif, Z. Gassara, S. Bendjmaa, M. Ezzeddine, M. H. Kallel, H. Fourati, R. Akrout, M. L. Masmoudi, S. Yaiich, S. Baklouti

**Affiliations:** 1 Rheumatology; 2 Epidemiology; 3 occupational medecine, Hedi Chalker Hospital, University of Sfax, Sfax, Tunisia

## Abstract

**Introduction:**

Ankylosing spondylitis (AS) is a chronic inflammatory disease that primarily affects the axial skeleton and may alter the quality of life of patients. Fatigue, one of the major clinical features of rheumatic diseases is a major clinical feature of AS, yet it has often been ignored in clinical practice.

**Objectives:**

This study aims to evaluate the quality of life in AS and to address the prevalence of fatigue in this disease and its associated factors.

**Methods:**

We conducted a cross-sectional study among AS patients. The Bath Ankylosing Spondylitis Functional Index (BASFI), the Bath Ankylosing Spondylitis Disease Activity Index (BASDAI), The Ankylosing Spondylitis Disease Activity Score (ASDAS) and other clinical measures were collected during the study.

We evaluated the psychometric characteristics of the Functional Assessment of Chronic Illness Therapy (FACIT)-Fatigue subscale) and the AS Quality of Life questionnaire (ASQoL). P values < 0.05 were considered statistically significant.

**Results:**

Sixty-two patients with AS were included in the study. The average age was 41 years [18-65]. The diagnostic delay was between 1 year and 26 years with an average of 4 years. The mean duration of the disease was 10 ± 8 years. The erythrocyte sedimentation rate (ESR) was between 2 and 50 mm/hour and the C reactive protein (CRP) level was between 1 and 45 mg/l. At baseline, the mean BASFI score was 53.9 ± 2, the mean BASDAI score was 4.5± 2 and the mean ASDAS score was 3.9 ± 2.

The mean FACIT-Fatigue score observed in these patients with AS was 20± 10,8 and the mean ASQoL score was 12,8 ± 4,8. Severe fatigue was observed in 43.5 % of patients and poor quality of life was detected in 62.9% of patients.

In univariate analysis, fatigue was associated with the low educational level of patients (p = 0.011), with sacroiliitis stage (p= 0.018) and with ASQoL score (p=0.000). ASQol was also associated with a high level of ESR (p=0.01). There was no relationship between FACIT or ASQoL and disease activity or functional status (p>0.05)

**Conclusions:**

This study confirms that poor QoL and fatigue were frequent in patients with AS which can expose them to the risk of psychiatric disorders like anxiety and depression. Therefore, patients suffering from AS should be regularly evaluated for these disorders.

**Disclosure of Interest:**

None Declared

